# Post-Transcriptional Regulation of RseA by Small RNAs RyhB and FnrS in *Escherichia coli*

**DOI:** 10.3389/fmolb.2021.668613

**Published:** 2021-11-03

**Authors:** Laricca Y. London, Joseph I Aubee, Jalisa Nurse, Karl M Thompson

**Affiliations:** ^1^ Department of Biological and Environmental Sciences, Alabama A&M University, Huntsville, AL, United States; ^2^ Department of Microbiology, College of Medicine, Howard University, Washington, DC, United States; ^3^ Department of Biology, Howard University, Washington, DC, United States

**Keywords:** small RNA, RyhB, FnrS, *Escherichia coli*, envelope stress response

## Abstract

RseA is the critical central regulator of the σ^E^-dependent stress response in *E. coli* and other related bacteria. The synthesis of RseA is controlled at the transcriptional level by several promoters and transcriptional regulators, including σ^E^ itself at two σ^E^-dependent promoters: *rpoE*_P_ and *rseA*_P3_. The presence of these two independent polycistrons encoding *rseA* is potentially redundant. We hypothesized that post-transcriptional control of the *rseA*_P3_ transcript was necessary to overcome this redundancy. However, to date, nothing is known about the post-transcriptional control of the *rseA*_P3_ transcript. We executed a targeted genetic screen to identify small RNA regulators of the *rseA*_P3_ transcript and identified RyhB and FnrS as small RNA activators of the RseA P3 transcript. Through genetic analysis, we confirmed that a direct interaction occurs between RyhB and RseA. We also identified sequences within the 5′ untranslated region (UTR) of RseA that were inhibitory for RseA expression. Point mutations predicted to prevent an interaction between RyhB and RseA resulted in increased RseA expression. Taken together, this suggests that the 5’ UTR of the RseAP3 transcript prevents optimal expression of RseA, preventing redundancy due to RseA expression from the σ^E^-dependent *rpoE*_P_, and this is overcome by the stimulatory activity of RyhB and FnrS.

## Introduction

The cell envelope promotes structural integrity of bacteria under dynamic conditions. The cell envelope can be perturbed *via* internal or external cues. Envelope stress occurs as a result of excess or mis-folded outer membrane proteins (OMPs), subsequently resulting in the activation of the extracytoplasmic function (ECF) sigma factor, σ^E^ (Erickson et al., 1987; Mecsas et al., 1993; Walsh et al., 2003). Over-expression or mis-folding of OMPs can compromise the integrity of the bacterial cell envelope and therefore bacterial cell survival (Walsh et al., 2003).

*E. coli* and other bacterial species have developed an envelope stress response (ESR) that restores homeostasis following the onset of envelope stress. The ESR can be mediated by several key regulators in *E. coli*, most notably CpxR and σ^E^ (Ravio, 1999; Ravio et al., 1999). In this work, we focused on the σ^E^-dependent ESR. Activation of σ^E^-dependent promoters results in the transcriptional initiation of at least 60 genes, many of which encode proteins necessary for the resolution of the ESR (Dartigalongue et al., 2001; Johansen et al., 2006; Rhodius et al., 2006; Thompson et al., 2007). Effectors of the ESR include small RNAs and periplasmic proteases that act to repress OMP levels (Douchin et al., 2006; Johansen et al., 2006; Rhodius et al., 2006; Thompson et al., 2007; Vogt et al., 2014). The stabilization of OMP levels restores cell envelope homeostasis. This also results in decreased σ^E^ activity. It is critically important to ensure that the steady-state levels of σ^E^ do not increase indefinitely as σ^E^ over-expression results in cell lysis and death (Nitta et al., 2000; Kabir et al., 2005). The small RNAs that act as σ^E^-dependent ESR effectors include MicA, RybB, and MicL; all of which have σ^E^-dependent promoters, repress expression of outer membrane proteins, and regulate LPS composition (Johansen et al., 2006; Thompson et al., 2007; Udekwu and Wagner, 2007; Klein et al., 2011; Klein and Raina, 2017).

**FIGURE 1:**
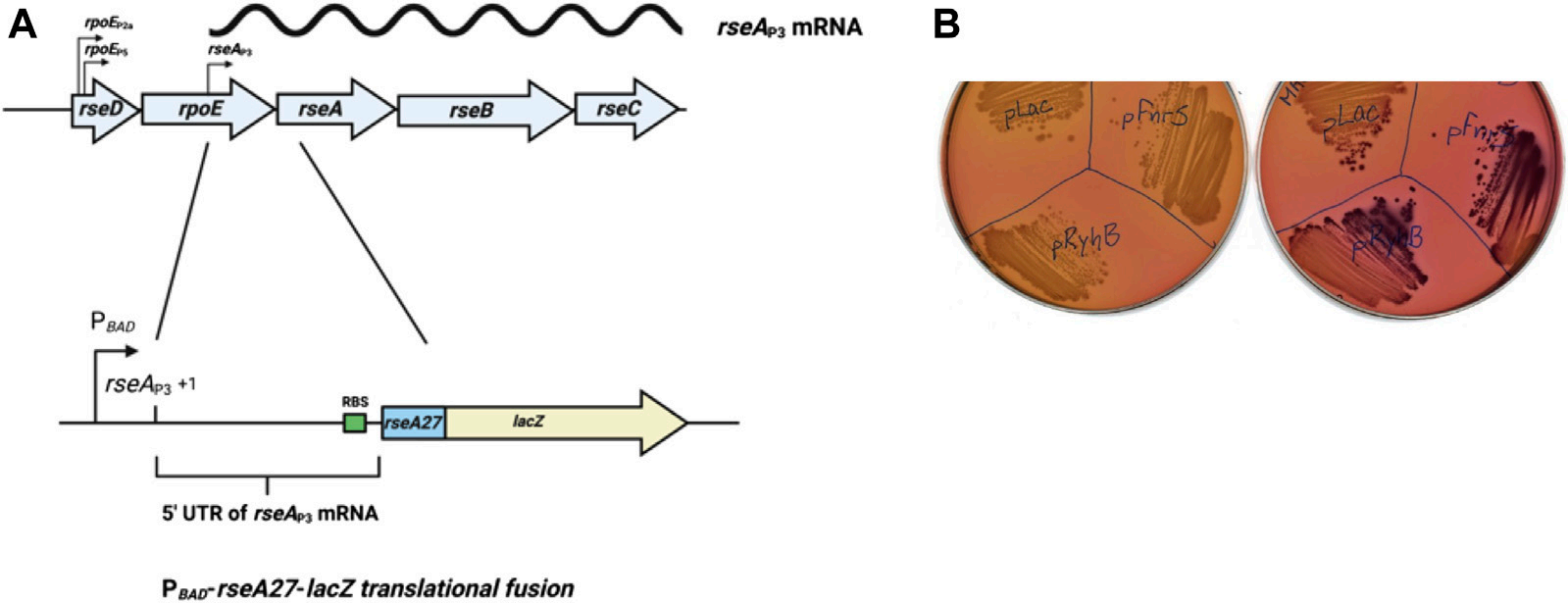
Genetic screen for small RNA regulators **(A)**. Schematic of the *rpoE* operon with selected promoters, including the *rseA*_P3_ promoter, and the P_BAD_-*rseA27-lacZ* translational fusion utilized for the small RNA library screen. The *rseA* fusion consists of the *rseA* P3 transcript, and the first nine codons of the *rseA* gene fused in the frame to the ninth codon of *lacZ*, downstream and controlled by the *araBAD* promoter (P_*BAD*_-*rseA27*-*lacZ* translational fusion) **(B)**. P_*BAD*_-*rseA27*-*lacZ* translational fusion strains transformed with an empty vector control (pBR-pLac), pBR-*ryhB*, or pBR-*fnrS* grown overnight at 37°C on MacConkey-Lactose agar plates. One plate was supplemented with 0.0002% arabinose to stimulate basal transcription of the P_*BAD*_-*rseA27*-*lacZ* translational fusion.

The σ^E^-dependent ESR is tightly controlled *via* regulation of the *rpoE* operon (*rseD*-*rpoE*-*rseA-rseB*-*rseC*) at transcriptional and post-transcriptional levels (Klein et al., 2016; Yakhnin et al., 2017). There are several promoters driving expression of this operon in concert with multiple transcription and sigma factors (Klein et al., 2016; Klein and Raina, 2017; Yakhnin et al., 2017). Transcriptional control of the σ^E^-dependent promoters is also controlled by guanosine 3′,5′-bispyrophosphate (ppGpp) and promoter architecture (Costanzo and Ades, 2006; Thompson et al., 2007; Costanzo et al., 2008; Balbontin et al., 2010). Only two of the promoters driving expression of the *rpoE* operon are σ^E^-dependent, while the others are cognate partners to σ^54^, σ^s^, σ^70^, or other transcriptional regulators (Klein et al., 2016). All promoters driving expression of the *rpoE* operon are localized upstream of, or within, the *rpoE* leader peptide (RseD) (Figure 1A). The remaining σ^E^-dependent promoter (*rseA*_P3_) is located within the *rpoE* coding region, over 230 nucleotides upstream of the *rseA* start codon (Rhodius et al., 2006). The σ^E^-dependent *rseA*_P3_ promoter drives the synthesis of an *rseA*-*rseB*-*rseC* transcript, while the σ^E^-dependent promoter *rpoE*_P_ drives the synthesis of the entire *rseD*-*rpoE*-*rseA*-*rseB*-*rseC* transcript (Rhodius et al., 2006). The activity of the σ^E^-dependent stress response is also regulated at the transcriptional level by the activity of RseA.

RseA is the anti-sigma factor for σ^E^ and plays a central role in envelope stress signal transduction (De Las Penans et al., 1997; Missiakas et al., 1997; Ades et al., 1999). RseA spans the inner membrane of the cell envelope and utilizes its cytoplasmic domain to interact with σ^E^ (Campbell et al., 2003). RseA sequesters σ^E^ to the inner member under vegetative growth conditions and thereby prevents access of σ^E^ to its cognate promoters (Campbell et al., 2003). Envelope stress stimulates a signal transduction cascade culminating in regulated intramembrane proteolysis (RIP) of RseA by periplasmic proteases DegS and RseP (De Las Penans et al., 1997; Missiakas et al., 1997; Alba et al., 2002; Kanehara et al., 2002). Following this, the N-terminus of RseA enters the cytoplasm in complex with σ^E^, whereby the RseA N-terminus is then cleaved by the adenosine 5′-triphosphate-dependent protease ClpXP (Flynn et al., 2004).

The synthesis of RseA from σ^E^-dependent *rseA*_P3_ and *rpoE*_P_ promoters has the potential to be physiologically redundant. It is likely that post-transcriptional regulation of *rpoE* and *rseABC* operons removes any physiological redundancy. While post-transcriptional regulatory mechanisms have been identified for the *rpoE* operon, little is known about post-transcriptional regulation of the *rseABC* operon. The *rseABC* operon has a 228 nucleotide 5′ untranslated region (UTR) (Rhodius et al., 2006). Due to the presence of the relatively long 5′ UTR for the *rseABC* operon, we hypothesized that *cis*-acting RNA structures and *trans*-acting small RNAs regulate the expression of *rseABC* operon. To test this hypothesis, we constructed an arabinose-inducible *rseA*-*lacZ* translational fusion and screened it with a small RNA library. We identified RyhB and FnrS as factors that stimulate post-transcriptional expression of RseA.

## Materials and Methods

### Media and Growth Conditions

All strains were grown in Luria Bertani (Lennox) liquid media (LB) at 37°C, with the exception of *λ*–Red-based recombineering strains using mini-*λ*:tet lysogens. These strains were grown in LB at 30°C and then shifted 43.5°C to induce expression of *λ*-Red proteins. Transformants were grown on LB agar plates supplemented with ampicillin, to a final concentration of 100 μg/ml. Zeomycin-resistant recombinants or transductants were selected on LB agar plates supplemented with Zeocin™ (or zeomycin) to a final concentration of 25 μg/ml. Small RNA screens were executed on MacConkey-Lactose (Mac-Lac) agar plates supplemented with ampicillin, to a final concentration of 100 μg/ml, and arabinose to a final concentration of 0.02% or 0.00002%. All gene fusions were created as previously described using recombineering and selecting for recombinant fusions on M63 minimal salt agar plates supplemented with glycerol, 6% sucrose, and 80 μg/ml of X-gal at 30°C (Mandin and Gottesman, 2009). For iron starvation experiments, cultures were grown in LB media in a shaking water bath at 37°C to an OD_600_ of 0.3 and then treated with 2′2-dipyridyl (Sigma Aldrich) to a final concentration of 250 μM for 30 min. For RNA stability assays, rifampicin was added to bacterial cultures to a final concentration of 250 μg/ml.

### Bacterial Strains, Plasmids, and Genetic Constructs

All strains used for experiments conducted in this study were derivatives of *Escherichia coli* K-12 MG1655. Cloning reactions were executed in MC1061 or NEB5α (New England Biolabs). All strains are listed in Table 1. All plasmids used in this study are listed in Table 2. All oligonucleotides used for polymerase chain reaction (PCR)-mediated genetic engineering, PCR screening, or Northern blot analysis are listed in Table 3. λ-Red recombineering reactions to gene fusions were executed in strain PM1800. PM1800 has a *cat*-*sacB* cassette inserted in the *lac* locus and encodes the λ-Red proteins (*gam*, *exo*, and *beta*) on a partial lambda vector marked with tetracycline resistance (mini-λ:*tet*). Plasmids from the small RNA library or their respective mutants were transformed into the P_*BAD*_-*rseA27*-*lacZ* translational fusion strain using TSS transformation (Chung et al., 1989). Mutations were transduced into reporter fusion strains using Bacteriophage P1 transduction.

**TABLE 1:** Strain list.

Strain	Genotype	Source
KMT1	*Escherichia coli* MG1655	
KMT432	MG1655 *lacI*::P_BAD_:*cat-sacB*:*lacZ*, Δ*araBAD*, *araC* ^+^, *mal*::*lacI* ^q^, minil::*tet* f80^+^	Pierre Mandin, Ph.D. (PM1205); Mandin and Gottesman (2009)
KMT465	MG1655 Δ*ryhB*::*zeo*	Nicolas DeLay, Ph.D
KMT467	MG1655 *lacI* ^q^ Δ*fnrS*::*kan*	Gisela Storz, Ph.D.; Durand and Storz (2010)
KMT519	NM525 pBR-pLac-*fnrS*-I	Giselia Storz, Ph.D.; Durand and Storz (2010)
KMT520	NM525 pBR-pLac-*fnrS*-II	Giselia Storz, Ph.D.; Durand and Storz (2010)
KMT521	NM525 pBR-pLac-*fnrS*-III	Giselia Storz, Ph.D.; Durand and Storz (2010)
KMT590	MG1655 *lacI*::P_BAD_:*cat-sacB*:*lacZ*, Δ*araBAD*, *araC*+, *mal*::*lacI* ^q^, minil::*tet* ϕ80^-^	Nadim Majdalani - PM1805
KMT20005	MG1655 Δ*araBAD*, *araC* ^+^, *mal*::*lacI* ^q^ *lacI*::*P*_*BAD*_ *-rseA27*-*lacZ* translational fusion	This work, KMT432 x *P*_*BAD*_ *rseA27*-*lacZ* PCR 42°C induction and selection on M63-Glycerol-XG
KMT20060	MG1655 Δ*araBAD*, *araC* ^+^, *mal*::*lacI* ^q^ *lacI*::*P*_*BAD*_-*rseA27*-*lacZ* translational fusion Δ*ryhB*::*zeo*	This work, KMT20005 x P1 (KMT465 - Δ*ryhB*::*zeo* source)
KMT20082	MG1655 Δ*araBAD*, *araC* ^+^, *mal*::*lacI* ^q^ *lacI*::P_*BAD*_-*rseA27*-*lacZ* translational fusion *(2)* Δ*ryhB*::*zeo* Δ*fnrS*::*kan*	This work, KMT20060 x P1 (KMT467 - Δ*fnrS*::*kan* source)
KMT20088	MG1655 Δ*araBAD*, *araC* ^+^, *mal*::*lacI* ^q^ *lacI*::P_*BAD*_-*rseA27*-*lacZ* translational fusion Δ*ryhB*::*zeo* Δ*fnrS*::*kan* pBR-pLac	This work, KMT20082 + pBR-pLac (TSS Transformation)
KMT20089	MG1655 Δ*araBAD*, *araC* ^+^, *mal*::*lacI* ^q^ *lacI*::P_*BAD*_-*rseA27*-*lacZ* translational fusion Δ*ryhB*::*zeo* Δ*fnrS*::*kan* pBR-pLac-*fnrS*	This work, KMT20082 + pBR-pLac-*fnrS* (TSS Transformation)
KMT20090	MG1655 Δ*araBAD*, *araC* ^+^, *mal*::*lacI* ^q^ *lacI*::P_*BAD*_-*rseA27*-*lacZ* translational fusion Δ*ryhB*::*zeo* Δ*fnrS*::*kan* pBR-pLac-*ryhB*	This work, KMT20082 + pBR-pLac-*ryhB* (TSS Transformation)
KMT20109	MG1655 Δ*araBAD*, *araC* ^+^, *mal*::*lacI* ^q^ *lacI*::P_*BAD*_-*rseA27*-*lacZ* translational fusion Δ*ryhB*::*zeo* Δ*fnrS*::*kan* pBR-pLac-*ryhBm*1	This work, KMT20082 + pBR-pLac-*ryhBm*1 (TSS Transformation)
KMT20110	MG1655 Δ*araBAD*, *araC* ^+^, *mal*::*lacI* ^q^ *lacI*::P_*BAD*_-*rseA27*-*lacZ* translational fusion Δ*ryhB*::*zeo* Δ*fnrS*::*kan* pBR-pLac-*ryhBm*2	This work, KMT20082 + pBR-pLac-*ryhBm*2 (TSS Transformation)
KMT20111	MG1655 Δ*araBAD*, *araC* ^+^, *mal*::*lacI* ^q^ *lacI*::P_*BAD*_-*rseA27*-*lacZ* translational fusion Δ*ryhB*::*zeo* Δ*fnrS*::*kan* pBR-pLac-*ryhBm*3	This work, KMT20082 + pBR-pLac-*ryhBm*3 (TSS Transformation)
KMT20112	MG1655 Δ*araBAD*, *araC* ^+^, *mal*::*lacI* ^q^ *lacI*::P_*BAD*_-*rseA27*-*lacZ* translational fusion Δ*ryhB*::*zeo* Δ*fnrS*::*kan* pBR-pLac-*ryhBm*4	This work, KMT20082 + pBR-pLac-*ryhBm*4 (TSS Transformation)
KMT20113	MG1655 Δ*araBAD*, *araC* ^+^, *mal*::*lacI* ^q^ *lacI*::P_*BAD*_-*rseA27*-*lacZ* translational fusion Δ*ryhB*::*zeo* Δ*fnrS*::*kan* pBR-pLac-*fnrS*-I	This work, KMT20082 + pBR-pLac-*fnrS*-I (TSS Transformation)
KMT20114	MG1655 Δ*araBAD*, *araC* ^+^, *mal*::*lacI* ^q^ *lacI*::P_*BAD*_-*rseA27*-*lacZ* translational fusion Δ*ryhB*::*zeo* Δ*fnrS*::*kan* pBR-pLac-*fnrS*-II	This work, KMT20082 + pBR-pLac-*fnrS*-II (TSS Transformation)
KMT20115	MG1655 Δ*araBAD*, *araC* ^+^, *mal*::lacI^q^ lacI::P_*BAD*_-*rseA27*-*lacZ* translational fusion Δ*ryhB*::*zeo* Δ*fnrS*::*kan* pBR-pLac-*fnrS*-III	This work, KMT20082 + pBR-pLac-*fnrS*-III (TSS Transformation)
KMT20144	MG1655 Δ*araBAD*, *araC* ^+^, *mal*::*lacI* ^q^ *lacI*::P_*BAD*_-*rseA27*-*lacZ* translational fusion	This work, KMT590 x P_*BAD*_-*rseA27*-*lacZ* gBlock amplified with KT902 + KT1137
KMT20146	MG1655 Δ*araBAD*, *araC* ^+^, *mal*::*lacI* ^q^ *lacI*::P_*BAD*_-*rseA27*cm1-*lacZ* translational fusion	This work, KMT590 x P_*BAD*_-*rseA27*cm1-*lacZ* gBlock amplified with KT902 + KT1137
KMT20148	MG1655 Δ*araBAD*, *araC* ^+^, *mal*::*lacI* ^q^ *lacI*::P_*BAD*_-*rseA27*cm3-*lacZ* translational fusion	This work, KMT590 x P_*BAD*_-*rseA27*cm3-*lacZ* gBlock amplified with KT902 + KT1137
KMT20156	MG1655 Δ*araBAD*, *araC* ^+^, *mal*::lacI^q^ *lacI*::P_*BAD*_-*rseA27*-*lacZ* cm1 Δ*fnrS*::*kan*	KMT20146 x P1 (Δ*fnrS*::*kan*)
KMT20160	MG1655 Δ*araBAD*, *araC* ^+^, *mal*::*lacI* ^q^ *lacI*::P_*BAD*_-*rseA27*-*lacZ* cm3 Δ*fnrS*::*kan*	This work, KMT20148 x P1 (Δ*fnrS*::*kan*)
KMT20172	MG1655 Δ*araBAD*, *araC* ^+^, *mal*::*lacI* ^q^ *lacI*::P_*BAD*_-*rseA27*cm1-*lacZ* cm1 Δ*fnrS*::*kan* Δ*ryhB*::*zeo*	This work, KMT20156 x P1 (Δ*ryhB*::*zeo*)
KMT20174	MG1655 Δ*araBAD*, *araC* ^+^, *mal*::*lacI* ^q^ *lacI*::P_*BAD*_-*rseA27*-*lacZ* cm3 Δ*fnrS*::*kan* Δ*ryhB*::*zeo*	This work, KMT20160 x P1 (Δ*ryhB*::*zeo*)
KMT20178	MG1655 Δ*araBAD*, *araC* ^+^, *mal*::*lacI* ^q^ *lacI*::P_*BAD*_-*rseA27*cm1-*lacZ* Δ*fnrS*::*kan* Δ*ryhB*::*zeo* pBR-pLac	This work, KMT20172 + pBR-pLac (TSS Transformation)
KMT20179	MG1655 Δ*araBAD*, *araC* ^+^, *mal*::*lacI* ^q^ *lacI*::P_*BAD*_-*rseA27*cm1-*lacZ* Δ*fnrS*::*kan* Δ*ryhB*::*zeo* pBR-pLac-*ryhB*	This work, KMT20172 + pBR-pLac-*ryhB* (TSS Transformation)
KMT20180	MG1655 Δ*araBAD*, *araC* ^+^, *mal*::*lacI* ^q^ *lacI*::P_*BAD*_-*rseA27*cm1-*lacZ* Δ*fnrS*::*kan* Δ*ryhB*::*zeo* pBR-pLac-*ryhBm*1	This work, KMT20172 + pBR-pLac-*ryhBm*1 (TSS Transformation)
KMT20184	MG1655 Δ*araBAD*, *araC* ^+^, *mal*::*lacI* ^q^ *lacI*::P_*BAD*_-*rseA27*cm3-*lacZ* Δ*fnrS*::*kan* Δ*ryhB*::*zeo* pBR-pLac	This work, KMT20174 + pBR-pLac (TSS Transformation)
KMT20185	MG1655 Δ*araBAD*, *araC* ^+^, *mal*::*lacI* ^q^ *lacI*::P_*BAD*_-*rseA27*cm3-*lacZ* Δ*fnrS*::*kan* Δ*ryhB*::*zeo* pBR-pLac-*ryhB*	This work, KMT20174 + pBR-pLac-ryhB (TSS Transformation)
KMT20186	MG1655 Δ*araBAD*, *araC* ^+^, *mal*::*lacI* ^q^ *lacI*::P_*BAD*_-*rseA27*cm3-*lacZ* Δ*fnrS*::*kan* Δ*ryhB*::*zeo* pBR-pLac-*ryhB*-m3	This work, KMT20174 + pBR-pLac-*ryhBm*3 (TSS Transformation)
KMT20196	MG1655 Δ*araBAD*, *araC* ^+^, *mal*::*lacI* ^q^ *lacI*::P_*BAD*_-*rseA*-3XFLAG w.t. #1	PM1800 x P_*BAD*_-*rseA*-3XFLAG w.t. amplified gBlock KT902 + KT1138
KMT20234	MG1655 Δ*araBAD*, *araC* ^+^, *mal*::*lacI* ^q^ *lacI*::P_*BAD*_-*rseA*-3XFLAG w.t. #1 Δ*ryhB*::*zeo*	KMT20196 x P1 (Δ*ryhB*::*zeo*)

**TABLE 2:** Plasmid list.

Plasmid	Characteristics	References or source
pBR-pLac	pBR322 ori (pMB1), P_LlacO_ promoter-based expression vector, *bla* (Amp^R^)	Guillier and Gottesman, 2006
pBR-pLac-*ryhB*	*ryhB* gene cloned into the AatII/EcoRI site of pBR-pLac	Mandin and Gottesman (2009)
pBR-pLac-*ryhBm*1	*ryhB* C14G, C15G, C16G site-directed mutations in pBR-pLac-*ryhB*	This work
pBR-pLac-*ryhBm*2	*ryhB* C18G, G19C, C20G, and G21C site-directed mutations in pBR-pLac-*ryhB*	This work
pBR-pLac-*ryhBm*3	*ryhB* G21C, G22C, A23C, G24A, A25C, and A26C site-directed mutations in pBR-pLac-*ryhB*	This work
pBR-pLac-*fnrS*	*fnrS* gene cloned into the AatII/EcoRI site of pBR-pLac	Mandin and Gottesman (2009)
pBR-pLac-*fnrS*-I	*fnrS* U57A, U58G, U59A site-directed mutations in pBR-pLac-*fnrS*	Gisela Storz, Durand and Storz (2010)
pBR-pLac-*fnrS*-II	*fnrS* C47A U48A U49G site-directed mutations in pBR-pLac-*fnrS*	Gisela Storz, Durand and Storz, (2010)
pBR-pLac-*fnrS*-III	*fnrS* G4C, G5U site-directed mutation in pBR-pLac-*fnrS*	Gisela Storz, Durand and Storz (2010)

**TABLE 3:** Oligonucleotide list.

Oligonucleotides	Sequence (5' - 3′)	Purpose
KT902	ACCTGACGCTTTTTATCGCAACTCTCTACTGTTTCTCCATGAGACAGATAGTTTTCCGAA	forward primer for amplification of the P_*BAD*_-*rseA27*-*lacZ* fusion recombination substrate
KT903	TAACGCCAGGGTTTTCCCAGTCACGACGTTGTAAAACGACTAAAGCGGAAAGTTGTTCTTTCTGCAT	reverse primer for amplification of the P_*BAD*_-*rseA27*-*lacZ* fusion recombination substrate
KT940	CGAAGCGGCATGCATTTACGTTG	Forward Screening primer for PM1205 fusions
KT950	CGTCGCGATCAGGAAGAGGGTCGCGGAGAACCTGAAA	forward primer for creation of *ryhBm*1 (C14G C15G C16G) by Quickchange mutagenesis
KT950c	TTTCAGGTTCTCCGCGACCCTCTTCCTGATCGCGACG	reverse primer for creation of *ryhBm*1 (C14G C15G C16G) by Quickchange mutagenesis
KT951	GCGATCAGGAAGACCCTGCGCGAGAACCTGAAAGCACG	forward primer for creation of *ryhBm*2 (C18G G19C C20G G21C) for Quickchange mutagenesis
KT951c	CGTGCTTTCAGGTTCTCGCGCAGGGTCTTCCTGATCGC	reverse primer for creation of *ryhBm*2 (C18G G19C C20G G21C) for Quickchange mutagenesis
KT952	GATCAGGAAGACCCTCGCCCCACCCCTGAAAGCACGACATTGGCACGACATTGCTCACCACACTTCCAGTATT	forward primer for creation of *ryhBm*3 (G22C G23C A24C G25A A26C A27C) for Quickchange mutagenesis
KT952c	AATACTGGAAGTGTGGTGAGCAATGTCGTGCCAATGTCGTGCTTTCAGGGGTGGGGCGAGGGTCTTCCTGATC	reverse primer for creation of *ryhBm*3 (G22C G23C A24C G25A A26C A27C) for Quickchange mutagenesis
KT953	GCACGACATTGCTCACCACACTTCCAGTATTACTTA	forward primer for creation of *ryhBm*4 (A51C T52A T53C G54A) for Quickchange mutagenesis
KT953c	TAAGTAATACTGGAAGTGTGGTGAGCAATGTCGTGC	reverse primer for creation of *ryhBm*4 (A51C T52A T53C G54A) for Quickchange mutagenesis
KT1115	CATTGACATTGTGAGCGGATAACAAGATACT	pBR-pLac forward screening and sequencing primer
KT1116	CCGCATTAAAGCTTATCGATGATAAGCTG	pBR-pLac reverse screening and sequencing primer
KT1137	CGCCAGGGTTTTCCCAGTCACGACGTTGTAAAACGACGGCTAAAGCGGAAAGTTGTTCTTTCTGCAT	reverse primer for amplification of the P_*BAD*_-*rseA27*-*lacZ* (wild-type and mutant) fusion recombination substrate

#### Construction of P_*BAD*_-*rseA27*-*lacZ* and Translational Fusion and P_*BAD*_-*rseA*-3XFLAG Strains

In order to execute our screen for small RNA regulation of the *rseABC* operon, we first created an arabinose-inducible in-frame translational fusion of the first nine codons of the *rseA* gene (*rseA27*) to the ninth codon of *lacZ via* recombineering into strain PM1800 as previously described (Mandin and Gottesman, 2009). We also created a 3XFLAG-tagged allele of the entire *rseA* gene at the lac locus using the same recombineering method (Mandin and Gottesman, 2009). All the fusions contained the entire 5′ UTR of the *rseABC* transcript (Figure 1A) immediately downstream from the arabinose-inducible *araBAD* promoter (P_*BAD*_). To create the allelic exchange substrates for either the P_*BAD*_-*rseA27*-*lacZ*, P_*BAD*_-*rseA27cm1*-*lacZ*, P_*BAD*_-*rseA27cm3*-*lacZ*, or P_*BAD*_-*rseA*-3XFLAG, or P_*BAD*_-*rseAcm1*-3XFLAG fusions, we amplified synthetic DNA gBlocks (IDT DNA) corresponding to each fusion using oligonucleotide primers KT902 and KT903. All synthetic DNA sequences used for genetic engineering of gene fusions are listed in Table 4. All oligonucleotide primers used for PCR reactions are listed in Table 3. We confirmed the presence of the *lacZ* translational fusion inserts by PCR using oligonucleotide primers KT940 and KT903 and DNA sequencing. We confirmed the presence of the 3XFLAG tagged alleles by PCR using oligonucleotide primers KT902 and KT1136 and DNA sequencing.

**TABLE 4:** List of synthetic DNA used in genetic engineering.

Sequence name	Sequence	Purpose
P_*BAD*_ *-rseA27-lacZ* gBlock	Acc​tga​cgc​ttt​tta​tcg​caa​ctc​tct​act​gtt​tct​cca​TGA​GAC​AGA​TAG​TTT​TCC​GAA​CTA​TTG​AGT​CCC​TCC​CGG​AAG​ATT​TAC​GCA​TGG​CAA​TAA​CCT​TGC​GGG​AGC​TGG​ATG​GCC​TGA​GCT​ATG​AAG​AGA​TAG​CCG​CTA​TCA​TGG​ATT​GTC​CGG​TAG​GTA​CGG​TGC​GTT​CAC​GTA​TCT​TCC​GAG​CGA​GGG​AAG​CTA​TTG​ATA​ACA​AAG​TTC​AAC​CGC​TTA​TCA​GGC​GTT​GAC​GAT​AGC​GGG​ATA​CTG​GAT​AAG​GGT​ATT​AGG​Cat​gCA​GAA​AGA​ACA​ACT​TTC​CGC​TTT​AGC​CGT​CGT​TTT​ACA​ACG​TCG​TGA​CTG​GGA​AAA​CCC​TGG​CG	used as a template for amplification of the recombination substrate for creation of P_*BAD*_ *-rseA27-lacZ* translational fusion
P_*BAD*_ *-rseA27cm1-lacZ* gBlock	Acc​tga​cgc​ttt​tta​tcg​caa​ctc​tct​act​gtt​tct​cca​TGA​GAC​AGA​TAG​TTT​TCC​GAA​CTA​TTG​AGT​CCC​TCC​CGG​AAG​ATT​TAC​GCA​TGG​CAA​TAA​CCT​TGC​GGG​AGC​TGG​ATG​GCC​TGA​GCT​ATG​AAG​AGA​TAG​CCG​CTA​TCA​TGG​ATT​GTC​CGG​TAG​GTA​CGG​TGC​GTT​CAC​GTA​TCT​TCC​GAG​CGA​CCC​AAG​CTA​TTG​ATA​ACA​AAG​TTC​AAC​CGC​TTA​TCA​GGC​GTT​GAC​GAT​AGC​GGG​ATA​CTG​GAT​AAG​GGT​ATT​AGG​Cat​gCA​GAA​AGA​ACA​ACT​TTC​CGC​TTT​AGC​CGT​CGT​TTT​ACA​ACG​TCG​TGA​CTG​GGA​AAA​CCC​TGG​CG	used as a template for amplification of the recombination substrate for creation of P_*BAD*_ *-rseA27(cm1)-lacZ* translational fusion
P_*BAD*_ *-rseA27cm3-lacZ* gBlock	Acc​tga​cgc​ttt​tta​tcg​caa​ctc​tct​act​gtt​tct​cca​TGA​GAC​AGA​TAG​TTT​TCC​GAA​CTA​TTG​AGT​CCC​TCC​CGG​AAG​ATT​TAC​GCA​TGG​CAA​TAA​CCT​TGC​GGG​AGC​TGG​ATG​GCC​TGA​GCT​ATG​AAG​AGA​TAG​CCG​CTA​TCA​TGG​ATT​GTC​CGG​TAG​GTA​CGG​TGC​GTT​CAC​GGA​GTG​GGC​GAG​CGA​GGG​AAG​CTA​TTG​ATA​ACA​AAG​TTC​AAC​CGC​TTA​TCA​GGC​GTT​GAC​GAT​AGC​GGG​ATA​CTG​GAT​AAG​GGT​ATT​AGG​Cat​gCA​GAA​AGA​ACA​ACT​TTC​CGC​TTT​AGC​CGT​CGT​TTT​ACA​ACG​TCG​TGA​CTG​GGA​AAA​CCC​TGG​CG	used as a template for amplification of the recombination substrate for creation of P_*BAD*_ *-rseA27(cm3)-lacZ* translational fusion
P_*BAD*_-*rseA*-*3XFLAG* gBlock	AcctgacgctttttatcgcaactctctactgtttctccaTGAGACAGATAGTTTTCCGAACTATTGAGTCCCTCCCGGAAGATTTACGCATGGCAATAACCTTGCGGGAGCTGGATGGCCTGAGCTATGAAGAGATAGCCGCTATCATGGATTGTCCGGTAGGTACGGTGCGTTCACGTATCTTCCGAGCGAGGGAAGCTATTGATAACAAAGTTCAACCGCTTATCAGGCGTTGACGATAGCGGGATACTGGATAAGGGTATTAGGCatgCAGAAAGAACAACTTTCCGCTTTAATGGATGGCGAAACGCTGGATAGTGAGCTGCTTAACGAACTGGCTCATAACCCAGAAATGCAGAAAACCTGGGAAAGCTATCACTTAATCCGTGACTCAATGCGGGGTGATACTCCCGAGGTGCTCCATTTCGATATCTCTTCACGCGTGATGGCCGCCATTGAAGAAGAGCCAGTACGTCAACCGGCGACATTGATCCCGGAAGCCCAGCCTGCGCCGCATCAATGGCAGAAAATGCCATTCTGGCAGAAAGTACGTCCGTGGGCGGCACAGCTTACCCAAATGGGCGTAGCCGCATGCGTATCGCTTGCAGTTATCGTTGGCGTCCAGCACTATAATGGACAATCTGAAACGTCCCAGCAGCCCGAAACGCCGGTATTTAATACACTGCCGATGATGGGTAAAGCCAGCCCGGTAAGCCTGGGAGTACCTTCTGAAGCGACCGCAAACAATGGTCAACAGCAGCAGGTACAGGAGCAGCGTCGTCGCATTAATGCAATGTTGCAGGATTACGAACTGCAACGCCGACTCCACTCTGAACAGCTTCAGTTTGAGCAGGCGCAAACCCAGCAAGCCGCTGTACAGGTGCCAGGAATTCAAACTTTAGGAACGCAATCGCAGGATTACAAAGATCATGACGGGGACTACAAAGATCACGATATAGATTATAAAGATGACGATGACAAAtaaATTATAAAAATTGCCTGATACGCTGCGCTTATCAGGCCTA	used as a template for amplification of recombination substrate for creation of P_*BAD*_-*rseA*-*3XFLAG*
P_*BAD*_-*rseAcm*1-*3XFLAG* gBlock	AcctgacgctttttatcgcaactctctactgtttctccaTGAGACAGATAGTTTTCCGAACTATTGAGTCCCTCCCGGAAGATTTACGCATGGCAATAACCTTGCGGGAGCTGGATGGCCTGAGCTATGAAGAGATAGCCGCTATCATGGATTGTCCGGTAGGTACGGTGCGTTCACGTATCTTCCGAGCGACCCAAGCTATTGATAACAAAGTTCAACCGCTTATCAGGCGTTGACGATAGCGGGATACTGGATAAGGGTATTAGGCatgCAGAAAGAACAACTTTCCGCTTTAATGGATGGCGAAACGCTGGATAGTGAGCTGCTTAACGAACTGGCTCATAACCCAGAAATGCAGAAAACCTGGGAAAGCTATCACTTAATCCGTGACTCAATGCGGGGTGATACTCCCGAGGTGCTCCATTTCGATATCTCTTCACGCGTGATGGCCGCCATTGAAGAAGAGCCAGTACGTCAACCGGCGACATTGATCCCGGAAGCCCAGCCTGCGCCGCATCAATGGCAGAAAATGCCATTCTGGCAGAAAGTACGTCCGTGGGCGGCACAGCTTACCCAAATGGGCGTAGCCGCATGCGTATCGCTTGCAGTTATCGTTGGCGTCCAGCACTATAATGGACAATCTGAAACGTCCCAGCAGCCCGAAACGCCGGTATTTAATACACTGCCGATGATGGGTAAAGCCAGCCCGGTAAGCCTGGGAGTACCTTCTGAAGCGACCGCAAACAATGGTCAACAGCAGCAGGTACAGGAGCAGCGTCGTCGCATTAATGCAATGTTGCAGGATTACGAACTGCAACGCCGACTCCACTCTGAACAGCTTCAGTTTGAGCAGGCGCAAACCCAGCAAGCCGCTGTACAGGTGCCAGGAATTCAAACTTTAGGAACGCAATCGCAGGATTACAAAGATCATGACGGGGACTACAAAGATCACGATATAGATTATAAAGATGACGATGACAAAtaaATTATAAAAATTGCCTGATACGCTGCGCTTATCAGGCCTA	used as a template for amplification of recombination substrate for creation of P_*BAD*_-*rseAcm1*-*3XFLAG*

#### Site-Directed Mutagenesis of pBR-pLac-*ryhB*

We used the QuikChange® Site-Directed Mutagenesis Kit (Stratagene), according to the manufacturer’s recommendations to create ryhB point mutants. Mutagenic primers used for the PCR reaction are listed in Table 3. Point mutants were verified by DNA sequencing.

### β-Galactosidase Assays

Overnight cultures were grown in Lennox Broth (LB) supplemented with ampicillin and glucose to a final concentration of 100 μg/ml and 0.2%, respectively, at 37°C. The cultures were diluted 1:1000 in fresh Lennox Broth (LB) supplemented with ampicillin and arabinose to a final concentration of 100 μg/ml and 0.02%, respectively. Once the culture reached an OD_600_ of 0.4–0.5, a 100 μL aliquot was taken for the β-galactosidase assay as previously described (Miller, 1992). Alternatively, β-galactosidase assays were executed in 96-well plates as previously described (Zhou and Gottesman, 1998).

### Western Blot Analysis

Cell lysates were created as previous described (Ezemaduka et al., 2014). Briefly, cells were harvested and resuspended in 300 ul of 20 mM Tris-HCl, pH 8.0, and lysed by sonication. The resulting cell lysates were centrifuged at 800 × *g* for 5-min to remove cell debris and unbroken cells. The supernatants for the respective samples were then transferred to new 1.5 ml tubes and subjected to centrifugation at 1,000 × *g* for 30 min. The resulting supernatant (soluble proteins) and precipitate (aggregated proteins) from the second centrifugation were then quantified using a Lowry assay and placed on ice. Equivalent amounts of total protein were prepared and subjected to electrophoresis using a Bolt™ 12%, Bis-Tris Protein Gel (Invitrogen), according to the manufacturer’s instructions. The total proteins were then transferred to a 0.45 μm pore-size nitrocellulose membrane using a Trans-Blot® Turbo™ System (BIO-RAD) for 10 min at 2.5 A and 25 V. After the successful transfer, the membrane was washed briefly in a phosphate-buffered saline and Tween 20 (PBST) solution and then blocked at room temperature for 30 min in 0.5% Blotting-Grade nonfat milk dissolved in PBST. The membrane was incubated overnight with gentle shaking at 4°C in a 1:50,000 dilution of a primary antibody and 0.5% Blotting-Grade nonfat milk blocking solution. The membrane was washed with PBST three times for 5 min each and incubated with gentle shaking in a 1:50,000 dilution of a secondary antibody and 0.5% Blotting-Grade nonfat milk blocking solution for 2 h. The signal was developed using a Novex AP Chemiluminescent Kit according to the manufacturer’s recommendations (Thermofisher Scientific). The protein signals were visualized using the FluorChem R imager (Protein Simple).

### Northern Blot Analysis

Total RNA was isolated using the hot acid phenol method as previously described (Aiba et al., 1981). RNA stability assays were performed as previously described using hot phenol following culture treatment with rifampicin to a final concentration of 250 µg/ml (Masse et al., 2003). Total RNA from each sample was mixed with the 10X RNA gel loading dye (National Diagnostics), 10X MOPS buffer, 100% formaldehyde, and 100% formamide and heated at 65°C for 15 min. The samples were loaded onto a 1% agarose gel and subjected to electrophoresis at 100 V for 40 min. The agarose gel was subjected to a nylon membrane capillary transfer. Following UV cross-linking, the membrane was pre-hybridized for 2 h using PerfectHyb™ Plus Hybridization Buffer (Sigma Aldrich). The membrane was then hybridized with a biotinylated DNA probe against RseA or 16S rRNA transcripts. (IDT DNA) for 4 h (Table 3). The membrane was washed with high, medium, and low stringency buffers and processed using a Chemiluminescent Detection Kit (Lifetechnologies) according to the manufacturer’s recommendation. The chemiluminescent signal was detected using Fluorochem E (Protein Simple).

### Statistical Analysis

All statistical analyses were executed using GraphPad Prism version 9 (GraphPad).

## Results

### RyhB and FnrS were Picked up in a Genetic Screen for Small RNA Regulators of RseA Expression

The start site of the *rseA*_P3_ promoter is 228 nucleotides upstream from the *rseA* start codon. The 228 nucleotide 5′ UTR likely forms a secondary structure that influences translational initiation in concert with small RNAs. We therefore hypothesized that the *rseABC* operon, driven by the *rseA*_P3_ promoter, was regulated at the post-transcriptional level by a small RNA. In order to determine if the *rseA*_P3_ transcript was regulated by a small RNA, we constructed an arabinose-inducible *rseA*-*lacZ* translational gene fusion (Figure 1A). We then transformed the P_BAD_-*rseA27*-*lacZ* translational fusion strain with a plasmid based small RNA library as previously described (Mandin and Gottesman, 2009). This small RNA library contains 30 of the most extensively characterized *E. coli* small RNAs cloned downstream of an isopropyl β-d-1-thiogalactopyranoside (IPTG)-inducible promoter (Mandin and Gottesman, 2009). We executed the small RNA library screen on MacConkey-Lactose (Mac-Lac) agar plates supplemented with ampicillin to a final concentration of 100 μg/ml and arabinose to a final concentration of 0.0002%. This arabinose concentration was sufficient to induce basal transcription from the P_BAD_ promoter without producing a strong Lac^+^ phenotype. The concentration of lactose in the Mac-Lac plates was sufficient to induce small RNA expression from the IPTG-inducible promoter. We hypothesized that this screening condition was ideal for the possible identification of stimulatory small RNAs. Two of the 30 plasmids, carrying RyhB or FnrS, resulted in an increased Lac phenotype (Figure 1B), suggesting that RyhB and FnrS promote post-transcriptional expression of RseA.

### RyhB and FnrS Point Mutants are Defective in Stimulating RseA Expression

**FIGURE 2:**
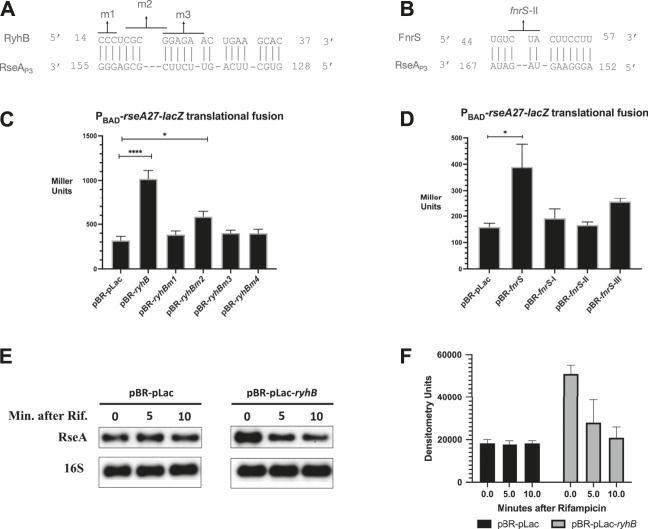
RyhB/FnrS pairing with RseA and β-galactosidase assays **(A)**. Regions of predicted base pairing between the RyhB and RseA sequences were determined using the online computational tool IntaRNA 2.0. Point mutants in the small RNA used for interaction analysis were denoted as m1 (C14G, C15G, C16G), m2 (C18G, G19C, C20G, and G21C), or m3 (G21C, G22C, A23C, G24A, A25C, and A26C) **(B)**. Δ*ryhB* Δ*fnrS* P_*BAD*_-*rseA27*-*lacZ* translational fusions containing pBR-pLac, pBR-*ryhB*, pBR-pLac-*ryhBm1*, pBR-pLac-*ryhBm2*, or pBR-pLac-*ryhBm3* were grown in rich media to an OD_600_ of 0.5, and aliquots were isolated for the β-galactosidase assay **(C)**. Regions of predicted base pairing between FnrS small RNA and the 5′ UTR of RseA were determined using the online computational tool IntaRNA 2.0. One of the *fnrS* point mutants tested occurs in the region of pairing shown **(B)**. Δ*ryhB* Δ*fnrS* P_*BAD*_-*rseA27*-*lacZ* translational fusions containing pBR-pLac, pBR-*ryhB*, pBR-pLac-*ryhBm1*, pBR-pLac-*ryhBm2*, or pBR-pLac-*ryhBm3* were grown in rich media to an OD_600_ of 0.5, and aliquots were isolated for the β-galactosidase assay **(D)**. Δ*ryhB* Δ*fnrS* P_*BAD*_-*rseA27*-*lacZ* translational fusions containing pBR-pLac, pBR-*fnrS*, pBR-pLac-*fnrS*-I, pBR-pLac-*fnrS*-II, or pBR-pLac-*fnrS-*III were grown in rich media to an OD_600_ of 0.5, and aliquots were isolated for the β-galactosidase assay **(E)**. P_*BAD*_-*rseA*27-lacZ translational fusions were grown to the mid-exponential phase and induced with arabinose and IPTG to a final concentration of 0.2% and 1 mM, respectively. Cultures were then treated with rifampicin, and total RNA was isolated at 5 and 10 min following rifampicin treatment. RNA was subjected to northern blot analysis using a biotinylated RseA probe **(F)**. Densitometric analysis of northern blot is shown in **Panel E**. All β-galactosidase assays and densitometry assays were executed in triplicate and are represented as averages ± the standard error of the mean (SEM). Statistical significance was assessed by one-way ANOVA with Dunnett’s post hoc test (*p < 0.05, ****p < 0.0001).

To validate the results of our genetic screen, we executed computational analysis to identify regions of potential complementary base pairing between RyhB or FnrS and the RseA leader region (Figures 2A,B). Specifically, we screened the RyhB and FnrS sequences against the RseA_P3_ 5′ UTR sequence using IntaRNA 2.0. For RyhB, we identified a semi-continuous stretch of nucleotides between C14 and C37 with complementarity to RseA_P3_ G128 to G155 (Figure 2A). The longest stretch of complementarity was seven base pairs between RyhB C14 to RyhB C20 and RseA G148 to G155. For FnrS, we identified a semi-continuous stretch of nucleotides between U44 and U57 with complementarity to RseA_P3_ A152 and A167 (Figure 2B). The longest stretch of which was a seven base pair region of complementarity between FnrS C51 to U57 and RseA A152 to G158 (Figure 2B). We then created nucleotide point mutants in the plasmid-based RyhB construct that would disrupt the predicted complimentary base pairing between RyhB and RseA (Figure 2A). The RyhB point mutants consist of the following changes: m1 (C14G, C15G, C16G), m2 (C18G, G19C, C20G, and G21C), and m3 (G21C, G22C, A23C, G24A, A25C, and A26C) (Figure 2A). The FnrS point mutants were previously described and consist of the following changes: *fnrS*-I (U57A, U58G, U59A), *fnrS*-II (C47A U48A U49G), and *fnrS*-III (G4C, G5U) (Durand and Storz, 2010).

In order to determine if the RyhB nucleotides predicted to pair with RseA are necessary for RseA stimulation, we determined the activity of the P_*BAD*_-*rseA27*-*lacZ* translational fusion upon over-expression of wild type and mutant alleles of RyhB. We executed these assays in a *ryhB* ^−^ *fnrS* ^−^ genetic background to ensure that the plasmids were the only source of RyhB or FnrS expression. We grew all strains in LB (Lennox) supplemented with ampicillin and arabinose to final concentrations of 100 μg/ml and 0.02%, respectively. We then obtained 100 μL aliquots of each culture in the mid-log phase of growth (OD_600_ of 0.5) and measured β-galactosidase activity. As expected, plasmid-based RyhB induced P_*BAD*_-*rseA27*-*lacZ* activity by approximately 3-fold in comparison to the vector control (Figure 2C), while all RyhB point mutants were defective for stimulation of P_*BAD*_-*rseA27*-*lacZ* (Figure 2C). This confirms the results of our genetic screen and suggests that RyhB stimulates post-transcriptional expression of RseA. Further, it suggests that RyhB nucleotides predicted to pair with nucleotides in the RseA leader region are necessary for RyhB post-transcriptional stimulation of RseA expression (Figure 2C). We executed a similar experiment using FnrS and a series of FnrS point mutants to determine if FnrS may stimulate the post-transcriptional expression of RseA. FnrS expression results in a 2-fold increase in P_*BAD*_-*rseA27*-*lacZ*. Each of the FnrS point mutants were also defective for stimulation of P_*BAD*_-*rseA27*-*lacZ*. To determine if the RyhB stimulatory effect on RseA_P3_ expression was due to an increase in RseA mRNA stability, we tested the stability of the RseA-LacZ following over-expression of RyhB (Figures 2E,F). We observed a 2-fold increase in RseA-LacZ mRNA levels prior to the initiation of mRNA stability measures. The change in RseA-LacZ mRNA levels is similar to the change in P_*BAD*_-*rseA27*-*lacZ* activity in the presence of RyhB. This suggests that RyhB stimulation of RseA post-transcriptional expression could occur at the level of transcription termination as previously described (Chen et al., 2019).

### Compensatory Mutations in RseA Suggest that RyhB Interacts with the RseA Leader Region

**FIGURE 3:**
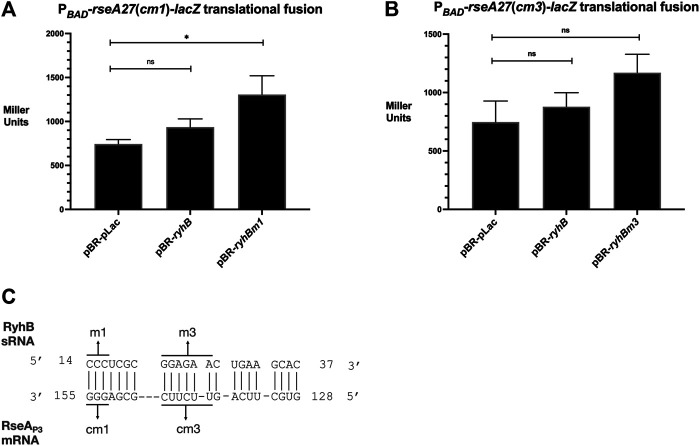
RyhB-RseA compensatory mutational analysis **(A)**. The P_*BAD*_-*rseA27*cm1-*lacZ* translational fusion strain transformed with pBR-pLac, pBR-pLac-*ryhB*, or pBR-pLac-*ryhBm1* was grown to the mid-log phase (OD_600_ of 0.5) in LB supplemented with ampicillin and arabinose to final concentrations of 100 μg/ml and 0.02%, respectively, and 100 μL aliquots were isolated for the β-galactosidase assay **(B)**. The P_*BAD*_-*rseA27*cm3-*lacZ* translational fusion strain transformed with pBR-pLac, pBR-pLac-*ryhB*, or pBR-pLac-*ryhBm3* was grown to the mid-log phase (OD_600_ of 0.5) in LB supplemented with ampicillin and arabinose to final concentrations of 100 μg/ml and 0.02%, respectively, and 100 μL aliquots were isolated for the β-galactosidase assay **(C)**. Putative nucleotide interactions between RyhB and RseA tested with RyhB mutants and RseA compensatory mutants. All β-galactosidase assays were executed in triplicate and are represented as averages ± SEM. Statistical significance was assessed by one-way ANOVA with Dunnett’s post hoc test (*p < 0.05).

In order to test the hypothesis that RyhB stimulates RseA through the direct interaction between complementary nucleotides, we designed putative compensatory nucleotide point mutations in the 5′UTR of P_*BAD*_-*rseA27*-*lacZ* translational fusions that were predicted to restore the ability of RyhB m1 or RyhB m3 mutants to stimulate P_BAD_-*rseA27*-*lacZ* activity (Figure 3C). The P_*BAD*_-*rseA27*(*cm1*)-*lacZ* translational fusion contains G153C G154C G155C nucleotide point mutants within the RseA 5′ UTR regions of the fusion, which were predicted to interact with the RyhB m1 allele (Figure 3C). The P_*BAD*_-*rseA27* (*cm3*)-*lacZ* allele contains U139G U141G C142U U143G U144G C145G nucleotide point mutants within the RseA 5′ UTR regions of the fusion, which were predicted to interact with the RyhBm3 allele (Figure 3C). RyhB over-expression does not result in the stimulation of cm1 or cm3 mutants of the P_*BAD*_-*rseA27*-*lacZ* fusion (Figures 3A,B). However, the expression of the RyhBm1 mutant was able to stimulate the P_*BAD*_-*rseA27*(*cm1*)-*lacZ* fusion, approximately 2-fold in comparison to the vector control (Figure 3A). In addition, the RyhBm3 allele was able to stimulate the P_*BAD*_-*rseA27*(*cm3*)-*lacZ* fusion, approximately 2-fold in comparison to the vector control (Figure 3B). These results suggest that RyhB activates post-transcriptional expression of RseA because of a direct interaction between RyhB and the RseA 5′ UTR. Furthermore, this interaction requires RyhB C14 C15 C16 nucleotides and the RseA 5′ UTR G153 G154 G155 nucleotides, as well as RyhB nucleotides G21, G22, A23, G24, A25, A26 and RseA nucleotides U139, U141, C142, U143, U144, C145 (Figure 3A).

### Optimal Post-Transcriptional Expression of RseA_P3_ is Inhibited by 5′ UTR Sequences

**FIGURE 4:**
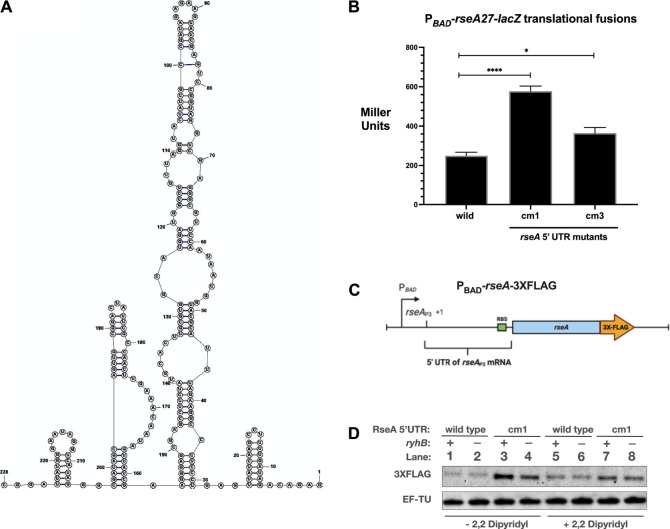
5′ UTR mutations stimulate RseA translation **(A)**. Predicted structure of RseA_P3_ 5′ UTR using RNAStructure **(B)**. The wild-type, cm1, and cm3 alleles of the P_*BAD*_ *-rseA27*-*lacZ* translational fusion strains were grown in rich media to the mid-log phase (OD_600_ of 0.5), and aliquots were isolated for β-galactosidase activity **(C)**. Schematic of the P_BAD_-*rseA*-3XFLAG allele utilized for the subsequent RseA-FLAG western blot **(D)**. RseA-FLAG western blot. The wild-type and cm1 P_*BAD*_-*rseA*-3XFLAG strains were grown in LB supplemented with arabinose to a final concentration of 0.002% to the mid-log phase and then treated with 2,2-dipyridyl to a final concentration of 250 μM. Total proteins were isolated and subjected to western blot analysis using α-FLAG. All β-galactosidase assays were executed in triplicate and are represented as averages ± SEM. Statistical significance was assessed by one-way ANOVA with Dunnett’s post hoc test (*p < 0.05, ****p < 0.0001).

Since the RseA_P3_ transcript is stimulated by RyhB and FnrS and RseA mRNA stability is not affected by RyhB over-expression, it is logical to assume that translation is repressed in the absence of the stimulatory small RNAs. Since the compensatory mutants in the RseA 5′ UTR subdued the ability of RyhB to stimulate RseA translation, we hypothesized that the cm1 and cm3 mutations would result in increased post-transcriptional expression of RseA in the absence of stimulatory RNAs RyhB and FnrS (Figures 2, 3A,B). The 5′ UTR sequence of the RseA_P3_ transcript was analyzed using the RNAStructure program. The predicted secondary structure contains several hairpins that may affect translation efficiency and accessibility of the RseA Shine Delgado sequence (Figure 4A). To test the hypothesis that the RseA compensatory mutant alleles have higher expression than wild-type alleles, we measured the activity of wild-type, cm1, and cm3 alleles of the P_*BAD*_-*rseA27*-*lacZ* translational fusion in the mid-log phase in rich media (Figure 4B). Both cm1 and cm3 alleles had higher β-galactosidase activity than the wild-type fusion (Figure 4B). The activity of the P_*BAD*_-*rseA27*(*cm3*)-*lacZ* translational fusion was approximately 3-fold higher than the activity of the wild-type P_*BAD*_-*rseA27*-*lacZ* translational fusion (Figure 4B). The activity of the P_*BAD*_-*rseA27* (*cm3*)-*lacZ* translational fusion was approximately 1.5–2-fold higher than the activity of the wild-type P_*BAD*_-*rseA27*-*lacZ* translational fusion (Figure 4B). We also created a chromosomal 3XFLAG epitope-tagged allele of *rseA*, driven by an arabinose inducible promoter, to measure post-transcriptional expression of the RseA_P3_ transcript in a manner that uncouples its synthesis from envelope stress (P_*BAD*_-*rseA*-3XFLAG at the *lac* locus–Figure 4C). We also created a cm1 allele of the P_*BAD*_-*rseA*-3XFLAG allele. This would allow us to determine if the RseA 5′ UTR mutations that increase P_*BAD*_-*rseA27*-*lacZ* activity correspond to increased RseA protein levels. We then grew (*ryhB* ^+^ and *ryhB* ^−^) wild-type and cm1 5′ UTR alleles of P_BAD_-*rseA*-3XFLAG in rich media supplemented with 0.002% arabinose to induce RseA-3XFLAG expression. At the mid-logarithmic phase, cultures were treated with 2,2-dipyridyl for 30 min to deplete iron and induce RyhB expression. We then isolated total protein and measured RseA-FLAG expression. RseA-FLAG levels were increased by at least 3-fold in the cm1 5′ UTR genetic background compared to the wild-type 5′ UTR genetic background (Figure 4D, lane 3 vs lane 1), consistent with our β-galactosidase assay results in Figure 4B. This further supports the idea that the wild-type sequence of the RseA_P3_ 5′ UTR contains secondary structures that prevent optimal translation. Also, the absence of *ryhB* in the cm1 5′ UTR genetic background resulted in a 3-fold decrease in RseA-FLAG protein levels from cells grown in rich media without 2,2-dipyridyl supplementation (Figure 4D, lane 4 vs lane 3). This suggests that the cm1 mutation is not sufficient for complete inhibition of RyhB stimulation of RseA expression. Unexpectedly, we did not see this difference in RseA-FLAG protein levels isolated from cells grown in rich media supplemented with 2,2-dipyridyl. In addition, the absence of *ryhB* did not change RseA-FLAG protein levels in the wild-type 5’ UTR genetic background for cells grown in rich media without 2,2-dipyridyl, whereby RyhB levels are not expected to be repressed by Fur. (Figure 4D, lanes 1 and 2). Unexpectedly, for reasons that are not clear, the absence of *ryhB* had no noticeable effect on the stimulation of RseA-FLAG protein levels under iron starvation conditions (Figure 4D, lanes 5 and 6).

## Discussion

Optimal levels of σ^E^ are critically important for a functional ESR. The absence of σ^E^ precludes the initiation of the ESR. Excess σ^E^ results in aberrant cell physiology and ultimately cell death (Nitta et al., 2000; Kabir et al., 2005). Fine tuning the synthesis and activity of σ^E^ is the major tool that the cell uses in order to achieve this goal. The *rpoE* operon includes several negative regulators of σ^E^ activity: *rseA*, *rseB*, and *rseC*, in addition to the *rpoE* leader peptide *rseD* (Klein et al., 2016). This particular operon has several promoters controlled by several transcriptional regulators and sigma factors, responding to a multitude of conditions (Klein et al., 2016). Since the negative regulators of σ^E^ are cistronic to *rpoE*, a negative feedback loop for σ^E^ activity is built into the synthesis of *rpoE*. The *rseA*_P3_ promoter drives the transcription of the *rseA*-*rseB*-*rseC* operon (Rhodius et al., 2006). Since this operon differs from the *rpoE* operon only in the absence of the *rseD* and *rpoE* genes, it suggests that secondary synthesis of the negative regulators of σ^E^ activity are necessary under conditions that are unique and separate from conditions driving the synthesis of the *rpoE* operon. The *rseA*_P3_ promoter and one of the several promoters of the *rpoE* operon are σ^E^-dependent. This highlights the regulatory redundancy in the σ^E^-dependent synthesis of RseA at the level of transcriptional initiation (Rhodius et al., 2006; Klein et al., 2016). This redundancy is likely reconciled through post-transcriptional regulation of *rpoE*_P_ and *rseA*_P3_ transcripts *via* mechanisms described in this work and additional regulatory switches that are undiscovered (Yakhnin et al., 2017). Genetic analysis of the P_*BAD*_-*rseA*27-*lacZ* translational fusion, RseA-FLAG protein levels from a P_*BAD*_-*rseA*-3XFLAG epitope-tagged allele, suggests that the wild-type sequence of the RseA_P3_ 5′ UTR prevents optimal RseA expression (Figure 3), presumably through RNA secondary structures that prevent efficient translation or promoting degradation of the RseA_P3_ transcript. This likely promotes tight control of RseA levels, specifically preventing excess of RseA levels in response to σ^E^ activity. However, the existence of the *rseA*_P3_ transcript driven by σ^E^-dependent promoters suggests that under specific conditions in concern with the σ^E^-dependent ESR, the cell requires the synthesis of additional RseA to prevent excess activity of σ^E^, which is deleterious in nature. The presence of a relatively long 5′ UTR, with a secondary structure suboptimal for the promotion of post-transcriptional expression, is an ideal cognate partner for one or more small regulatory RNAs. While a direct interaction with RseA is necessary for the post-transcriptional regulatory effect of RyhB, its precise molecular mechanism is not clear but a part of an ongoing investigations in our lab. The difference in RyhB-dependent RseA-LacZ mRNA decay rates suggests that a complicated mechanism of action may occur. Possible pathways for the RyhB regulatory effect on RseA include modulation of translation initiation or transcription termination.

We identified RyhB and FnrS as small RNAs with positive regulatory effects on RseA synthesis using a targeted genetic screen of a small RNA library. FnrS expression is induced in response to oxygen limitation (Durand and Storz, 2010). RyhB is expressed under iron-limiting conditions upon de-activation of the Fur repressesor (Masse and Gottesman, 2002). RyhB was also previously identified as a binding partner for RseA using the RNA interaction by ligation and sequencing (RIL-seq) (Melamed et al., 2016). RyhB is known to repress post-transcriptional expression of a host of genes, including iron sulfur cluster proteins involved in the tricarboxylic acid cycles, in response to iron limitation (Masse and Gottesman, 2002; Masse et al., 2003; Semsey et al., 2006; Desnoyers and Masse, 2012; Wright et al., 2013). There is at least one positive regulatory target for RyhB, the shikimate permease gene ShiA (Prevost et al., 2007). FnrS has several regulatory targets, all of which are negative regulatory targets (Boysen et al., 2010; Durand and Storz, 2010; Wright et al., 2013). The observations in this work expand the RyhB regulon by adding a second positive regulatory target. This work also expands the FnrS regulon by uncovering the first positive regulatory target of FnrS. RyhB and FnrS share three unique targets, SodA, SodB, and MarA (Masse and Gottesman, 2002; Afonyushkin et al., 2005; Boysen et al., 2010; Durand and Storz, 2010; Argaman et al., 2012; Wright et al., 2013). In addition, both RyhB and FnrS have post-transcriptional regulatory targets in the *iscR*-*iscS*-*iscU*-*iscA* operon (Desnoyers et al., 2009; Wright et al., 2013). Our observation of both RyhB and FnrS regulating RseA is consistent with previously reported overlapping targets for RyhB and FnrS. RyhB and RseA are highly conserved in Gram-negative bacteria. It is possible that RyhB homologues or other small RNAs in these bacterial species regulate the post-transcriptional synthesis of RseA homologues.

For reasons that are not clear, there may be an increased requirement for RseA synthesis when iron, or oxygen, limitation occurs simultaneously with envelope stress. In our studies, we observed an increase in RseA-FLAG protein levels under iron-limiting conditions, implicating iron starvation in the post-transcriptional expression of the RseA_P3_ transcript. In the cm1 genetic background, RseA-FLAG protein levels decrease in the absence of RyhB (Figure 4D, lanes 3 vs 4). This supports our over-expression studies and implicate RyhB in the post-transcriptional stimulation of the RseA_P3_ expression. However, it is puzzling that the *ryhB* mutant did not prevent an increase in RseA levels under iron-limiting conditions (Figure 4D, Lanes 1/2 vs Lanes 5/6). It is possible that the change in RseA protein levels is transient in nature. Further investigated is needed to fully ascertain the physiological link between iron limitation, RyhB, and RseA expression. However, it is clear that both iron limitation and chromosomal RyhB levels affect post-transcriptional expression of the RseA_P3_ transcript. This also further suggests that excess σ^E^ activity may be especially deleterious to the cell under iron limitation. There have been some studies executed in *E. coli* and *Vibrio sp*. that support a link between envelope stress and iron limitation. Under iron-limiting conditions, suboptimal secretion of the siderophore enterobacterin results in the induction of the Cpx-dependent ESR (Guest et al., 2019). The Cpx-dependent ESR links iron sensing and adaptation in *Vibrio cholerae* (Acosta et al., 2015). The treatment of *Vibrio vulnificus* with the broad-spectrum antibiotic tropodithietic acid (TDA) simultaneously induced the expression of genes involved in cell envelope biogenesis, oxidative stress, and iron limitation (Dittman et al., 2019). Treatment of *V. cholerae* with polymyxin B results in induction of the σ^E^-dependent stress response and iron metabolism changes (Sikora et al., 2009). From studies in these systems, there appears to be a link between the iron metabolism and envelope stress. The precise mechanisms linking the iron metabolism and envelope stress are the subject of ongoing investigations in our lab.

## Data Availability

The raw data supporting the conclusion of this article will be made available by the authors, without undue reservation.
